# Responses of phyllosphere microbiota and plant health to application of two different biocontrol agents

**DOI:** 10.1186/s13568-019-0765-x

**Published:** 2019-03-28

**Authors:** Chong Qin, Jiemeng Tao, Tianbo Liu, Yongjun Liu, Nengwen Xiao, Tianming Li, Yabing Gu, Huaqun Yin, Delong Meng

**Affiliations:** 1 School of Minerals Processing and Bioengineering, Central South University, Changsha, China; 2 Key Laboratory of Biometallurgy, Ministry of Education, Central South University, Changsha, China; 3 Tobacco Research Institute of Hunan Province, Changsha, China; 4 College of Agronomy, Hunan Agricultural University, Changsha, China; 5 State Key Laboratory of Environmental Criteria and Risk Assessment, Chinese Research Academy of Environmental Sciences, Beijing, China

**Keywords:** Biocontrol agent, Phyllosphere microbiota, Tobacco wildfire disease, Community diversity, Molecular ecology networks

## Abstract

The phyllosphere supports a tremendous diversity of microbes, which have the potential to influence plant biogeography and ecosystem function. Although biocontrol agents (BCAs) have been used extensively for controlling plant diseases, the ecological effects of BCAs on phyllosphere bacteria and the relationships between phyllosphere community and plant health are poorly understood. In this study, we explored the control efficiency of two BCA communities on bacterial wildfire disease by repeatedly spraying BCAs on tobacco leaves. The results of field tests showed that BCAs used in our study, especially BCA_B, had remarkable control effects against tobacco wildfire disease. The higher control efficiency of BCA_B might be attributed to a highly diverse and complex community in the phyllosphere. By 16S ribosomal RNA gene sequencing, we found that phyllosphere microbial community, including community diversity, taxonomic composition and microbial interactions, changed significantly by application of BCAs. According to the correlation analysis, it showed that wildfire disease infection of plants was negatively related to phyllosphere microbial diversity, indicating a highly diverse community in the phyllosphere might prevent pathogens invasion and colonization. In addition, we inferred that a more complex network in the phyllosphere might be beneficial for decreasing the chances of bacterial wildfire outbreak, and the genera of *Pantoea* and *Sphingomonas* might play important roles in wildfire disease suppression. These correlations between phyllosphere community and plant health will improve our understanding on the ecological function of phyllosphere community on plants.

**Electronic supplementary material:**

The online version of this article (10.1186/s13568-019-0765-x) contains supplementary material, which is available to authorized users.

## Introduction

Bacterial pathogens are associated with plant diseases and can account for major economic losses to agricultural production. The management of plant diseases in the sustainable agriculture has become a challenge for plant pathologist. Several strategies have been recommended to control disease incidence and severity, such as chemical pesticides and biological control (Erwin and Ribeiro 1996). However, the use of chemical pesticides often results in environmental and food quality problems (Sharma et al. 2012). As an ecologically viable alternative, biological control has been a desirable strategy for controlling plant diseases (You et al. 2015) and there are an increasing number of biocontrol agents (BCAs), such as *Bacillus* spp., *Pseudomonas* spp., *Trichoderma* spp. etc., being commercialized for various crops (Trabelsi and Mhamdi 2013; Cha et al. 2016).

Disease suppression by BCAs is the manifestation of interactions among the plant, the pathogen, the biocontrol agents, the microbial community on and around the plant, and the physical environment (Akhtar and Siddiqui 2010). BCAs were selected from nonpathogenic or antagonistic microorganisms, and applied to foliar or root tissues of plants (Jones et al. 2012). Wildfire disease is a serious disease on tobacco leaves (Venkategowda et al. 2013). The pathogen, *Pseudomonas syringae* pv. *tabaci*, colonizes on tobacco surfaces before infection, and the size of the resultant populations is correlated with the severity of wildfire disease (Rouse et al. 1985). The biocontrol of plant root disease can be obtained by manipulating the rhizosphere microflora by favoring beneficial microorganisms (Janvier et al. 2007; Santhanam et al. 2015). Therefore, the control of wildfire disease could be more direct and effective by applying BCAs on the leaves. The aerial part of plants (the phyllosphere) is also an important and ubiquitous habitat for diverse community of microorganisms (Vorholt 2012). Compared to studies on soil microbiota, studies of phyllosphere microbiota have been rare and mainly focused on fruit and vegetable crops (Jensen et al. 2013; Leff and Fierer 2013). Studies have demonstrated that phyllosphere communities also have potential for plant biogeography and ecosystem function (Meyer and Leveau 2012) by producing growth-promoting compounds (Reed et al. 2010) or by protecting hosts against pathogen infection (Innerebner et al. 2011). Although the importance of phyllosphere bacteria on plants is well recognized, the ecological effects of BCAs on phyllosphere bacteria and the relationships between phyllosphere community and plant health are complex and poorly understood.

The phyllosphere is an open system and phyllosphere microorganisms are completely exposed to the atmosphere (Mueller and Ruppel 2014). Therefore, phyllosphere communities could be easily affected by exogenous factors, such as UV radiation, air pollution and biological inoculation (Williams et al. 2013). Inoculation BCAs can alter the indigenous microorganisms within the phyllosphere, thereby affecting the community’s ecological and functional properties (Zhang et al. 2008). For example, by introducing *Trichoderma harzianum* T22 to the phyllosphere of strawberries, the fungal composition and diversity showed a great change at class level, whereas the bacterial composition and diversity were not affected (Sylla et al. 2013b). By means of plate counts and 454 pyrosequencing, Sylla et al. (2013a) also presented the community changes of strawberries phyllosphere after application of *Aureobasidium pullulans* during two subsequent years. The microbial changes, in some cases, might affect sustainable plant production and plant health (Sang and Kim 2012). However, in addition to diversity and composition of the community, microbial interactions are also vital parts of the microbiome and the interactions are important for determining the ecosystem functioning (Zhou et al. 2011). Our previous study has demonstrated that a more complex soil ecology network may help suppress tobacco wilt (Yang et al. 2017). Therefore, the response of microbial interactions within the phyllosphere to BCAs may also be an important aspect to assess the efficacy of BCAs.

The aim of the present study is to evaluate the effects of two foliar BCAs on phyllosphere microbiota of tobacco and further to reveal the potential relationships between phyllosphere community and plant health. Therefore, two BCA communities, which were screened independently against tobacco wildfire disease in previous work, were introduced to the tobacco field every 7 days for four times. During this process, we investigated the infection rate and disease index of plant, detected the structural changes of phyllosphere microbial community by sequencing of 16S rRNA gene amplicons, and constructed molecular ecological networks based on random matrix theory (RMT). As a result, the present study offers an integrated insight into the relationships between phyllosphere microbial community and tobacco wildfire disease.

## Materials and methods

### Experimental design

Two biocontrol agents (BCAs, namely BCA_A and BCA_B) were used in the present study. The agents were screened from the healthy tobacco leaves through antagonistic tests by the team of our laboratory. The healthy leaves were collected from the field with both healthy and infected plants in Longshan County, China. In the antagonistic and pot experiments, the agents have shown high biocontrol potential against *Pseudomonas syringae* pv. *tabaci*. After batch fermentation in LB culture medium, the cell density of the agents reached 1.25 × 10^12^ CFU (Colony-Forming Units) L^−1^. The sequencing data of the agents have been made publicly available in the Sequence Read Archive (SRA) database of NCBI following the accession number of PRJNA515831. Results of sequencing showed that the BCA_A agent was mainly consisted with 21 different genera (Additional file 1: Fig. S1) with *Stenotrophomonas* (49.45%), *Achromobacter* (22.92%), *Enterobacter* (14.25%), *Ochrobactrum* (10.05%), and *Pseudomonas* (2.72%) as abundant (> 1%) genera, whereas the BCA_B agent was dominated by *Bacillus* (87.74%), *Alcaligenes* (7.69%), *Pseudochrobactrum* (2.86%) and *Achromobacter* (1.05%). Statement: For request of the BCAs, please contact the corresponding author (Dr. Meng) (For research purpose only).

The growth periods of tobacco in the field include transplanting seedling stage, root extending stage, vigorous growth stage and maturing stage. Wildfire disease of tobacco often occurs and spread widely in the vigorous growth stage. Therefore, the biocontrol agents were applied in this period. The field experiment was conducted in 2017 at Xiangxi Tobacco Test Base (109°25′E, 29°14′N, el. 672 m) in Longshan County, Hunan Province, China. Tobacco plants (K326) were transplanted to a 275.4 m^2^ region (16.2 m × 17 m) with a strain spacing of 0.6 m and a row spacing of 1.0 m on May 10th 2017. The experiment included three treatments, which were arranged in a randomized complete block design with 12 plots (each treatment including four replications), and the plot size was 18 m^2^ (Additional file 1: Fig. S2). The plants were irrigated with 300 kg ha^−1^ water after being transplanted. During the growth period, the agricultural management practices and fertilization regimes were similar in all plots. Fertilizer was applied according to previous practice (Xiao et al. 2018) and no measures were done for pest and disease control, except for the treatments described below.

From June 28th 2017, tobacco plants were sprayed with BCA_A and BCA_B weekly for four times. Fermented agents (about 1.25 × 10^12^ CFU L^−1^) was diluted with sterile deionized water to 1% (vol/vol) suspensions that contained bacterial cells at 10^7^ CFU mL^−1^ (Wei et al. 2016). Tobacco leaves in each treatment were sprayed with 20 L diluted suspensions via a hand-held sprayer each time. Control plants were treated with equal amount of sterile deionized water.

### Incidence estimating, sample collection and microbe elution

Eight plants were labeled randomly and used for the assessments of wildfire disease incidence in each plot. Disease infection rate and disease index of wildfire disease were recorded on June 28th (before BCAs application), July 12th (14 days after BCAs application) and July 26th (28 days after BCAs application), respectively. Disease infection rate was calculated according to the following equation:

1 $${\text{Infection rate }}\left( \% \right)\, = \,\left( {{{n_{i} } \mathord{\left/ {\vphantom {{n_{i} } {n_{t} }}} \right. \kern-0pt} {n_{t} }}} \right)\, \times \, 100$$

where *n*_*i*_ is the number of infected tobacco plant and *n*_*t*_ is the total number of tobacco plant in each plot.

Disease index was used to represent the mean disease rating of tobacco and calculated by the following equation:

2 $${\text{Disease index }}\left( \% \right)\, = \,\left[ {\sum {{{\left( {r \times n_{i} } \right)} \mathord{\left/ {\vphantom {{\left( {r \times n_{i} } \right)} {\left( {n_{t} \times R} \right)}}} \right. \kern-0pt} {\left( {n_{t} \times R} \right)}}} } \right]\, \times \, 100$$

where *r* is the degree of disease infection, *n*_*i*_ is the number of infected tobaccos corresponding to the grade of *r*, *n*_*t*_ is the total number of tobaccos tested, and *R* is the value of the highest degree of disease infection among the tested plants. The degrees of disease infection (*r*) were assigned to six grades (0, 1, 3, 5, 7, 9) as previously described (Alamri et al. 2019).

When assessing disease incidence, two leaf samples from the eight labeled plants in each field replicate (8 leaf replicates for each treatment) were randomly collected and transferred into plastic bags. In laboratory, 10 g samples from each tobacco leaf were used for microbe elution. Microbes were eluted by shaking leaves in phosphate-buffered saline (PBS) buffer. The detailed operational methods were described in previous protocols (Sylla et al. 2013b; Wei et al. 2016). Then, the mixtures were centrifugated at a slow speed (5 min, 500 rpm, 4 °C) to remove leaf residues and the remaining supernatant was centrifuged at 10,000 rpm for 15 min at 4 °C. Microorganisms were collected from the precipitate and frozen at − 20 °C before DNA extraction.

### DNA extraction, PCA amplification, sequencing and data preprocessing

DNA extraction, amplification of 16S rRNA amplicons and sequencing were performed as described in our previous studies (Tao et al. 2016, 2018). Briefly, DNA was extracted using MoBio PowerSoil DNA Isolation Kits (MO BIO, San Diego, CA), and then was used as template to amplify the V4 region of 16S rRNA gene with the primer pair 515F (5′-GTGCCAGCMGCCGCGGTAA-3′) and 806R (5′-GGACTACHVGGGTWTCTAAT-3′) primers (Caporaso et al. 2012). After purification, PCR products were used for library construction and sequenced by the Illumina MiSeq platform (Illumina, San Diego, CA). Sequences processing was conducted on the Galaxy pipeline (http://zhoulab5.rccc.ou.edu:8080/root) according to our previous description (Tao et al. 2016, 2018). After quality trimming, the pair-end reads were combined with at least 10-bp overlap and lower than 5% mismatches through Flash (Magoc and Salzberg 2011). The combined sequences were handled with the removal of shorter sequences and chimeras and were assigned to operational taxonomic unit (OTU) at 97% similarity level by UPARSE (Edgar 2013). The taxonomy of OTU sequences was performed through RDP Classifier (Wang et al. 2007) at a 50% confidence threshold. All the 16S rRNA gene sequences were deposited in the NCBI Sequence Read Archive (SRA) database, and the project number is PRJNA512544.

### Data analysis

All calculations and statistical analyses for microbial community were carried out using R software (version 3.4.0). Shannon-Weiner’s index (H), Simpson index (D), Pielou evenness (E) and Inverse Simpson diversity (Inv-D) indexes, were calculated with package ‘vegan’ (Dixon 2003). The observed OTU number was calculated by counting the observed OTUs in each sample. The Chao1 diversity index was calculated as the following equation that Chao1 = *Sobs* + *F*_*1*_^2^/*2F*_*2*_, Where *Sobs* is the observed OTU number, *F*_*1*_ is the number of singletons and *F*_*2*_ is the number of doubletons. Phylogenetic diversity of phyllosphere microbial communities from different treatments was compared using the indexes of mean nearest taxon distance (MNTD), mean phylogenetic distance (MPD), net relatedness index (NRI) and nearest taxon index (NTI). The values of MNTD, MPD, NRI and NTI were calculated using the ‘picante’ packages in R.

Community structure of the phyllosphere was measured by the analyses of principal coordinates analysis (PCoA) and non-metric multidimensional scaling (NMDS), which were carried out with ‘vegan’ package based on weighted Unique Fraction of branches shared (UniFrac) distances (Lozupone et al. 2011). Dissimilarity analyses were used to detect the significance level of the differences in bacterial community composition between groups (i.e., different stages and different treatments). Therefore, multi-response permutation procedure (MRPP), analysis of similarity (ANOSIM) and permutational multivariate analysis of variance (PERMANOVA or ADONIS) were also performed using ‘vegan’ package based on Bray–Curtis distance matrix.

The molecular ecological network (MEN) was constructed based on OTU relative abundance in different treatments. The steps of network construction were referenced to a previous study (Zhou et al. 2011). In the present study, only the OTUs present in at least 5 out of 8 biological replicate samples were kept for network construction. Random matrix theory (RMT) was used to choose the similarity threshold (*St*) automatically before network construction (Zhou et al. 2010; Deng et al. 2016). All network analyses were performed at a public web server (http://129.15.40.240/MEAN/). Gephi 0.9.1-beta (Bastian et al. 2009) was used to visualize the network interactions. Modules in each network were randomly colored.

The graphs and charts were generated by Origin 9.0 or R v. 3.4.0. One-way analysis of variance (ANOVA) followed by the Tukey’s test was used to measure the differences between treatments at the same time in SPSS 22.0 (SPSS Inc., Chicago, USA). A *p* value of less than 0.05 represents the significant difference. All experiments were performed at least three times.

## Results

### Plant health

**Fig. 1:**
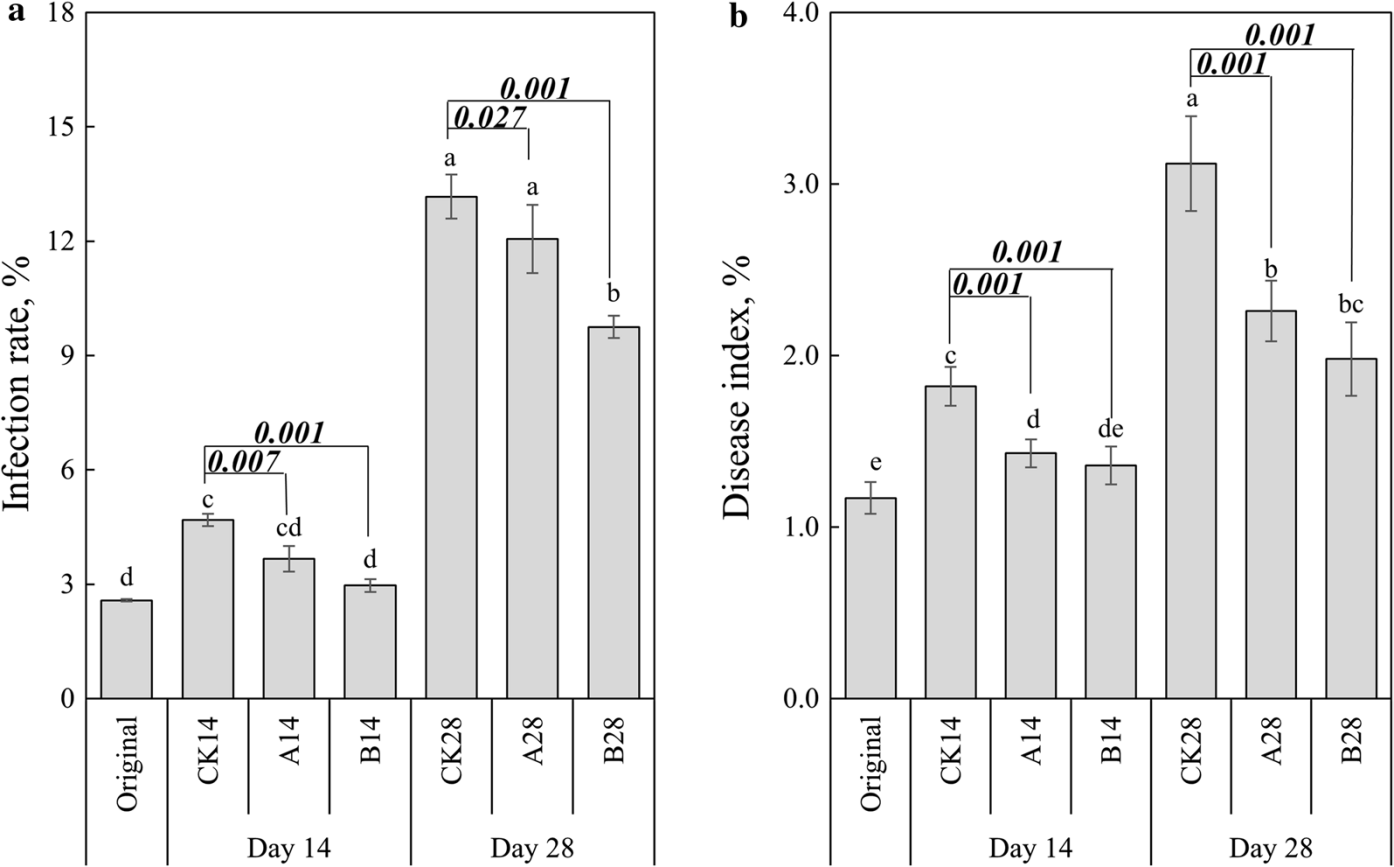
Plant disease infection rate and disease index. Results are means and SD (error bar) of four replicates, statistically significant differences (*p* < 0.05) in values between treatments are indicated through different letters above the columns, numbers between two columns are p values of student *t*-test

Disease infection rate and disease index were used to describe the wildfire disease infection level. The disease infection rate and disease index showed similar trends among all the treatments (Fig. 1). Compared to the control, significant decreases were observed for both disease infection rate and disease index in BCA applicated treatments, at both Day 14 and Day 28. At Day 28, the disease infection rate in BCA_B applicated field was significantly lower than BCA_A. The results indicated that the BCAs, especially BCA_B, played positive roles in inhibiting the wildfire disease.

### Overall responses of phyllosphere microbial communities to BCAs

Phyllosphere microbial communities were analyzed by sequencing 16S rRNA V4 gene amplicons with Illumina Miseq. A total of 3,039,004 high quality sequences were obtained across all samples. To remove any differences caused by sequencing depth, all samples were rarefied by randomly choosing 20,000 sequences, and the rarefied OTU table was used for further analyses. The rarefaction curves (Additional file 1: Fig. S3) showed that increasing the sequencing depth would not lead to obvious increase in OTU numbers, indicating the sequencing is adequate for downstream analysis. From this sequencing data, 2574 OTUs were clustered with 97% identity and 480 genera and 38 phyla were identified from this data when blast the sequences in RDP database (Additional file 1: Fig. S4). The phyllosphere microbial community was dominated by the phylum *Proteobacteria* that averagely account for 88% of the microbial community, while at the genus level, *Pseudomonas, Sphingomonas, Pantoea, Streptophyta, Tatumella,* and *Acinetobacter* were the abundant genera that had an average relative abundance of more than 1%. The bacterial composition of the phyllosphere by application of BCAs was apparently different at genus levels with plant growing. For example, at Day 14, the most abundant genus in control was *Sphingomonas*, whereas *Pseudomonas* was the dominant genus in BCAs treatments. However, at Day 28, *Pseudomonas* accounted for more than 92% in control group, *Streptophyta* was more abundant in BCA_A treatment, and *Sphingomonas* was significantly higher in BCA_B treatment. Besides, *Pantoea* always accounted for a higher proportion in both BCA_A and BCA_B treatments at both Day 14 and Day 28.

**Fig. 2:**
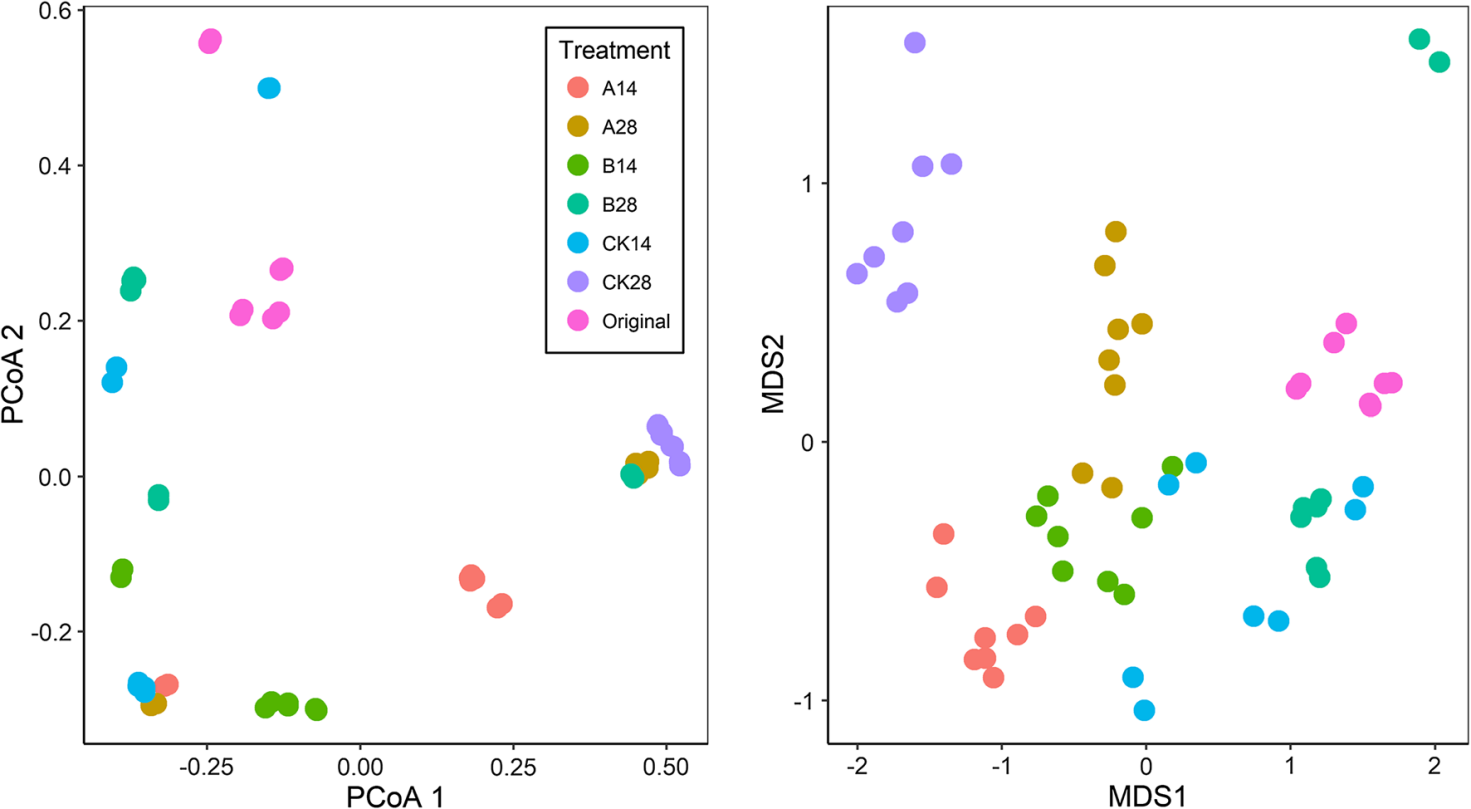
Taxonomic beta-diversity of phyllosphere microbial community as indicated by principal coordinates analysis (PCoA) and non-metric multidimensional scaling (NMDS) plots

The taxonomic α-diversity indices including observed OTU number (Sob), Chao1, Shannon (H), Inverse Simpson (Inv-D), Simpson (D) and Pielou evenness (E) were used to judge the variation of microbial community diversity. All taxonomic α-diversity indices showed similar trends among treatments (Table 1). The taxonomic α-diversity indices showed obvious differences among stages or between treatments. When no BCAs were applicated, the taxonomic α-diversity indices decreased with time, whereas t-test showed that application of BCAs led to significant increase in microbial community diversity. Obvious differences were observed in the microbial community composition between treatments at both phylum and genus level as shown in Additional file 1: Fig. S4. PCoA and NMDS analyses further showed that phyllosphere microbial community structure (at OTU level) of different stages or different treatments (i.e. control, BCA_A and BCA_B) was clearly divided (Fig. 2). Statistical analyses including ANOSIM, ADNOIS and MRPP based on taxonomic Bray–Curtis distance further confirmed that the microbial community with different treatments varied significantly (Additional file 1: Table S1).

**Table 1:** Taxonomic diversity of phyllosphere microbial communities

	Sob	Chao1	H	Inv-D	D	E
Original	504 ± 127a	619 ± 123a	3.08 ± 1.22a	12.49 ± 10.61a	0.77 ± 0.27a	0.49 ± 0.18a
CK14	286 ± 101b	448 ± 159b	1.90 ± 0.38bc	3.25 ± 0.93b	0.66 ± 0.13ab	0.34 ± 0.07bc
A14	136 ± 43c*	252 ± 91bc*	2.35 ± 0.44ab*	5.65 ± 1.7b*	0.81 ± 0.07a*	0.48 ± 0.06ab*
B14	170 ± 33c*	349 ± 66c	2.14 ± 0.18bc	5.66 ± 1.37b*	0.81 ± 0.06a*	0.42 ± 0.05ab*
Ck28	69 ± 8c	134 ± 29c	0.42 ± 0.16d	1.18 ± 0.09b	0.15 ± 0.07c	0.10 ± 0.04d
A28	170 ± 16c*	278 ± 42c*	1.28 ± 0.56 cd*	2.26 ± 1.32b	0.45 ± 0.20b*	0.25 ± 0.11c*
B28	444 ± 58a*	715 ± 62a*	2.16 ± 0.52bc*	3.64 ± 1.31b*	0.68 ± 0.15ab*	0.36 ± 0.08abc*

Results are means and SD of replicates (n = 8)

*Sob* observed OTU number, *H* Shannon diversity index, *Inv-D* inverse Simpson diversity index, *D* Simpson diversity index, *E* Pielou evenness

Statistically significant differences (*p* < 0.05) in values between treatments are indicated through different letters

The ‘*’ indicates the difference between treatment A or B and control (at the same day) is significant at *p* < 0.05 level as assessed by student *t*-test

**Fig. 3:**
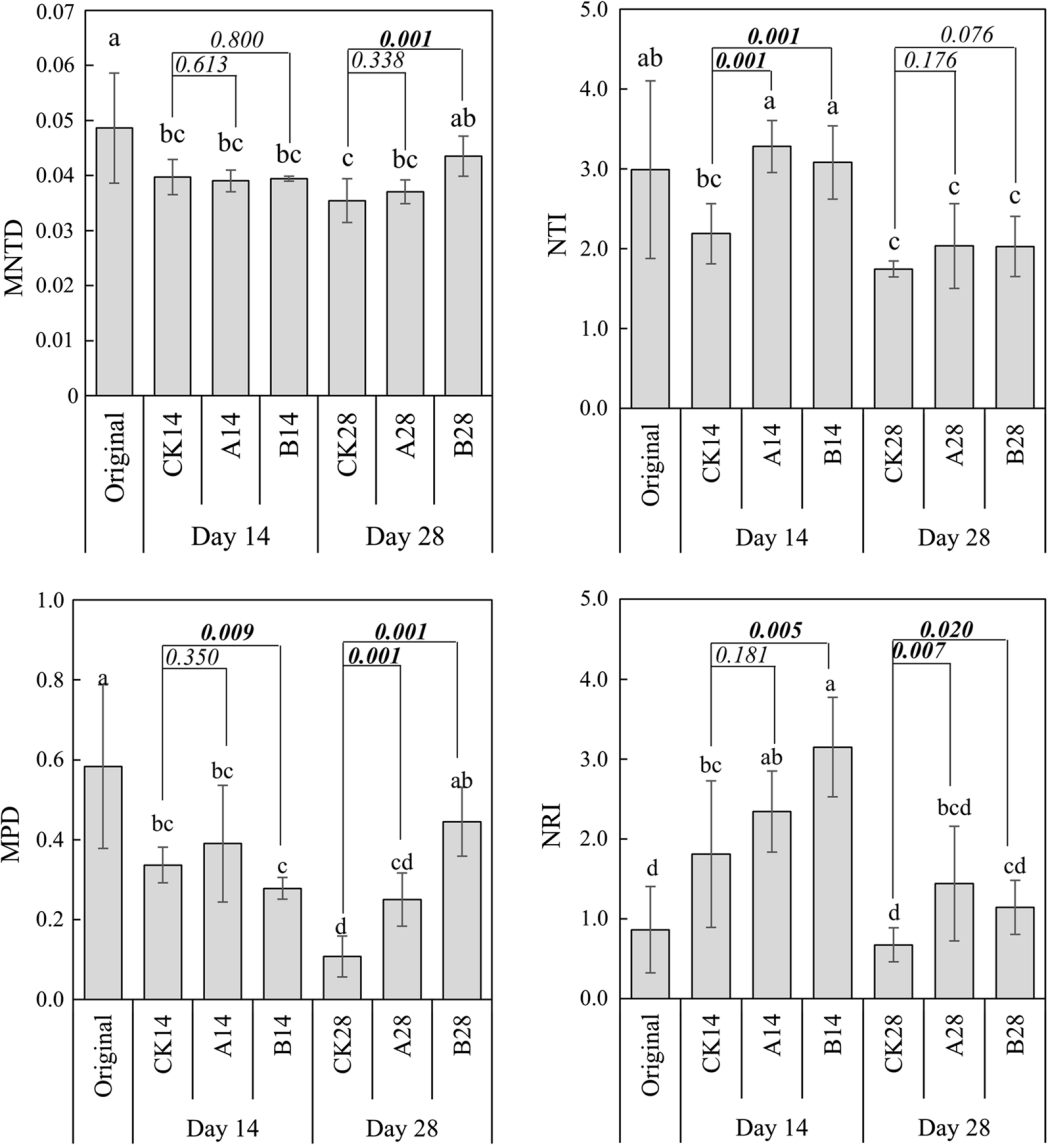
Phylogenetic diversity of phyllosphere microbial communities. *MNTD* mean nearest taxon distance, *NTI* near taxon index, *MPD* mean phylogenetic distance, *NRI* net relatedness index. Results are expressed as means and SD (error bar) of 8 replicates, statistically significant differences (*p* < 0.05) in values between treatments are indicated through different letters above the columns, numbers between two columns are *p* values of student *t*-test

**Fig. 4:**
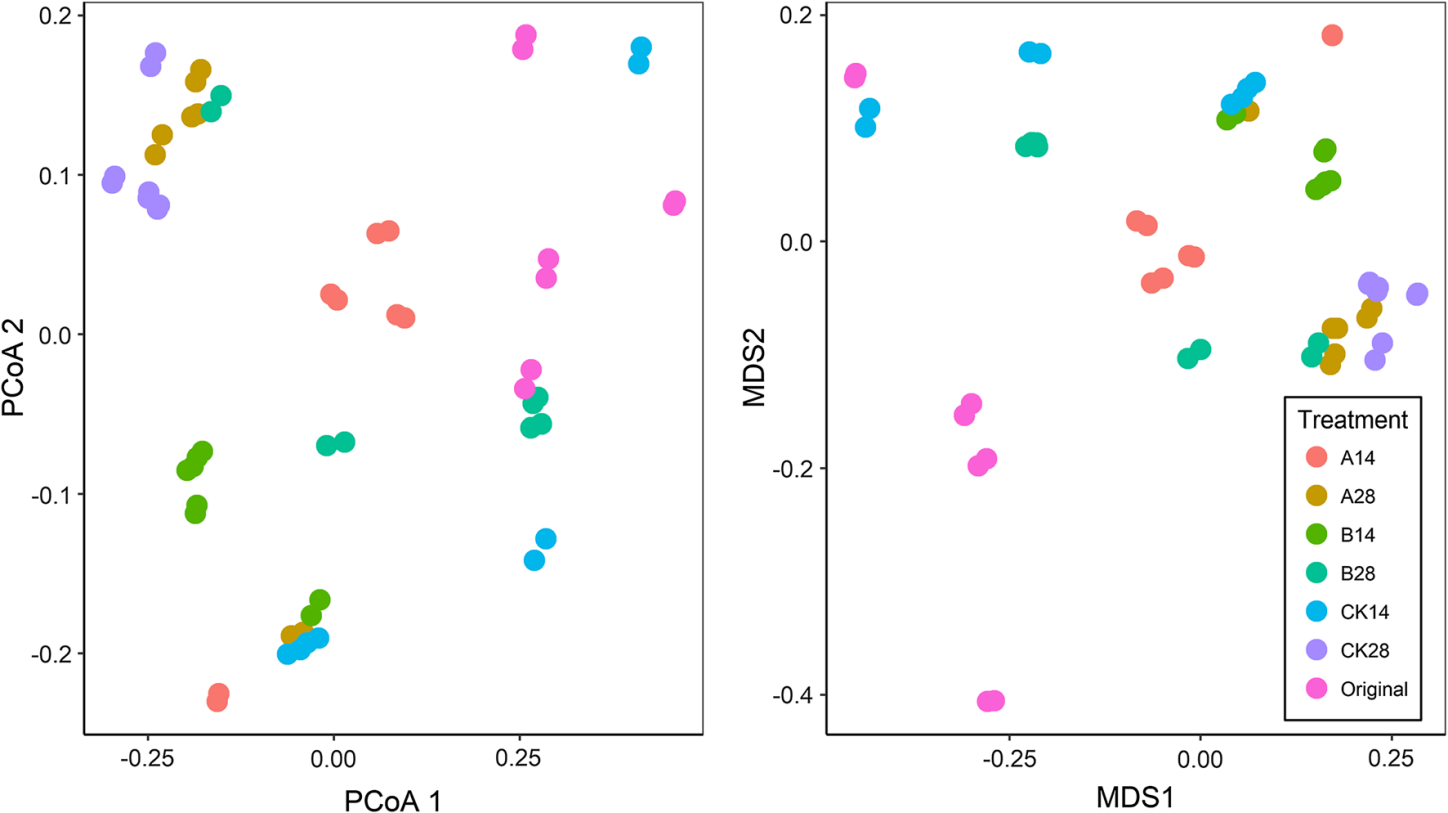
Phylogentic beta-diversity of phyllosphere microbial community as indicated by principal coordinates analysis (PCoA) and non-metric multidimensional scaling (NMDS) plots

Phylogenetically, microbial community diversity was described by mean-nearest-taxon-distance (MNTD), nearest-taxon-index (NTI), mean phylogenetic distance (MPD) and net relatedness index (NRI) (Webb et al. 2002) (Fig. 3). The values of MNTD, NTI and MPD decreased significantly (*t*-test, *p* < 0.05) with plant growing (original vs CK14 vs CK 28). For the MNTD, application of BCAs did not cause any significant changes at Day 14, whereas, at Day 28, the MNTD was significantly (*t*-test, *p* < 0.05) higher by application of BCA_B. The NTI obtained using the null model were significantly positive (which means the standardized effect size of MNTD values were negative), indicating phyllosphere microbial communities tended to be more phylogenetically clustered than would be expected by chance. Application of BCAs significantly increased (*t*-test, *p* < 0.05) the NTI at Day 14, whereas, at Day 28, there were no differences between different treatments. For the MPD and NRI, application of BCA_B significantly (*t*-test, *p* < 0.05) decreased the MPD and increased the NRI at Day 14, whereas, at Day 28, the MPD and NRI were both significantly (*t*-test, *p* < 0.05) higher by application of BCAs. When refer to the phylogenetic beta-diversity of phyllosphere microbial community, PCoA and NMDS based on phylogenetic distance showed microbial communities of different treatments were clearly separated (Fig. 4). The dissimilarity analysis including ANOSIM, ADNOIS and MRPP indicated the differences between treatments or between stages were significant (Additional file 1: Table S2).

### Random matrix theory-based molecular ecology network

**Fig. 5:**
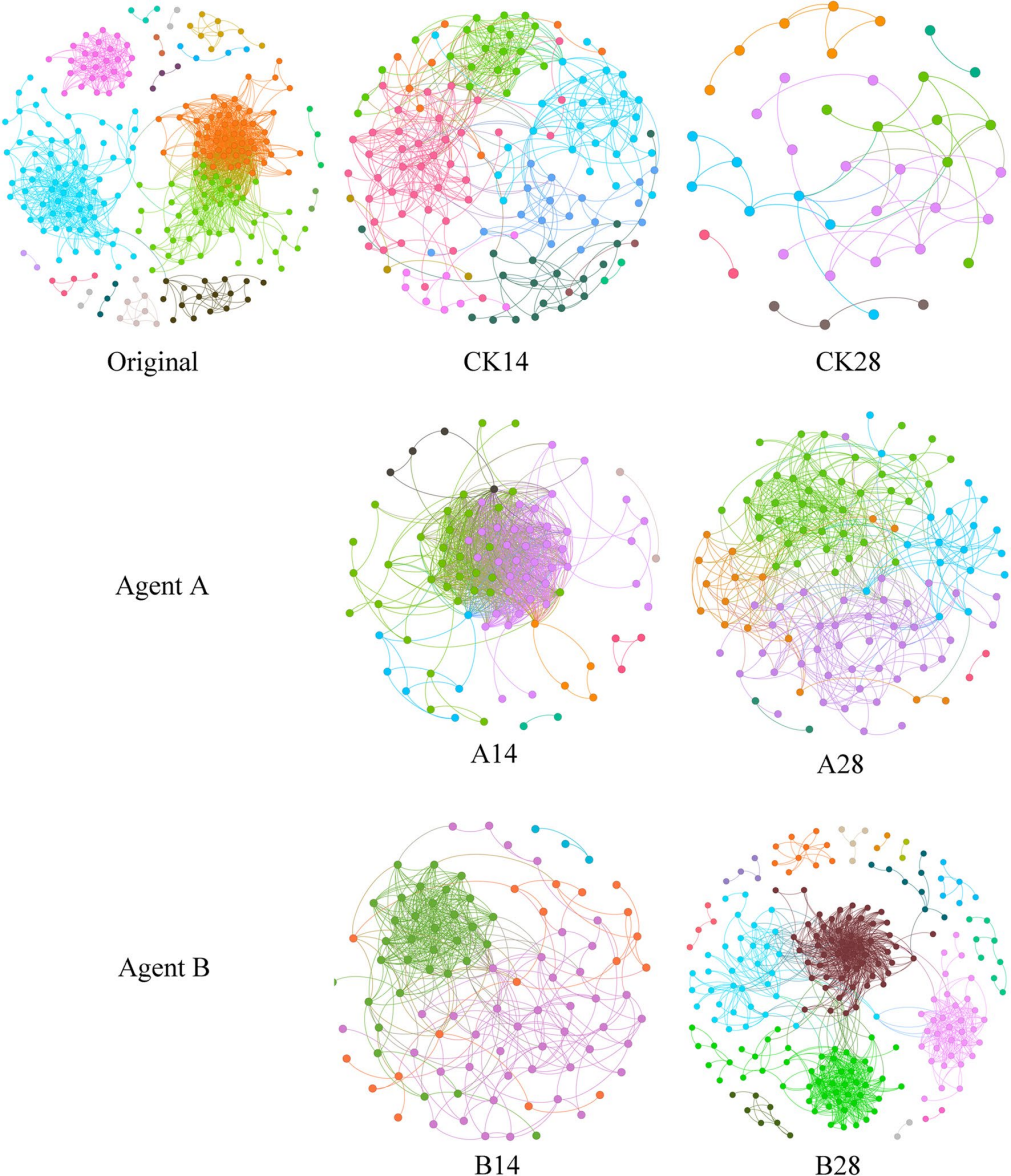
Random matrix theory (RMT) based molecular ecology network. Networks represent random matrix theory models derived from 8 biological replicates in each cropping system. Each node represents an OTU. The links between the nodes indicate strong and significant (*p* < 0.01) correlation. Modules are presented with different colors

Based on the 16S rDNA sequence data of phyllosphere bacterial communities, RMT-based network analysis method was used to discern the variation of potential microbe–microbe interactions under the two BCAs application. The phyllosphere networks differed profoundly among plant growth stages (Original vs CK14 vs CK28 in Fig. 5) and different treatments (CK24 vs A28 vs B28 in Fig. 5). The networks became simple in control over time but became connected and complex when application of BCA_B. Multiple network topological properties also indicated the similar variation between networks (Additional file 1: Table S3). When no BCAs were applicated, the number of nodes, links and average degree decreased significantly (*p *< 0.05) with time, whereas application of BCA_B led to the increase of nodes and links, indicating the increased network complexity. At Day 28, the phyllosphere network of BCA_B application contained 1 414 links among 236 nodes, which was much higher than the networks of control (55 links) and BCA_A application (602 links) (Additional file 1: Table S3), reflecting a higher number of microbial co-occurrence in phyllosphere community by application of BCA_B.

### Correlation between phyllosphere microbial community and plant health

**Fig. 6:**
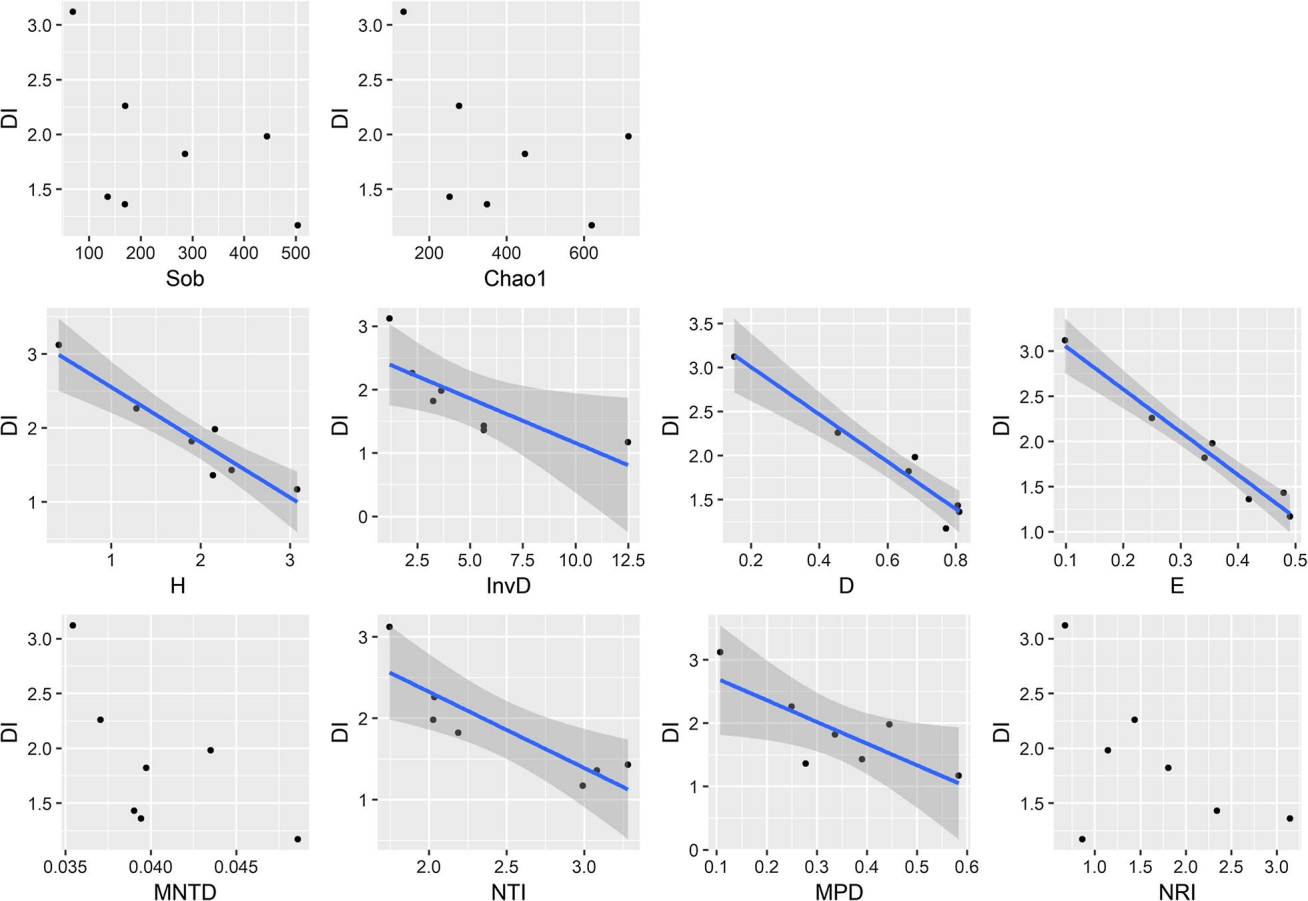
Correlation between plant health and the characteristics of phyllosphere microbial community. *DI* disease index, *Sob* observed OTU number, *Chao1* Chao1 richness index, *H* Shannon diversity index, *InvD* inverse Simpson diversity index, *D* Simpson diversity index, *E* Pielou evenness, *MNTD* mean nearest taxon distance, *NTI* near taxon index, *MPD* mean phylogenetic distance, *NRI* net relatedness index. The blue lines and shades represent the regression lines with 95% confidence intervals. Pearson correlation indexes and *p*-values are shown in Additional file 1: Table S4

The correlation analyses were performed between diversity indexes (taxonomic and phylogenetic diversity) of phyllosphere community, network properties and disease index of wildfire disease (Fig. 6 and Additional file 1: Table S4). For taxonomic diversity, disease index did not show any significant associations with either observed OTU number (Pearson = − 0.494, *p* = 0.260) or Chao1 richness (Pearson = − 0.493, *p* = 0.261), but exhibited significantly negative correlations with other four taxonomic diversity indexes, including Shannon diversity index (Pearson = − 0.947, *p* = 0.001), Inverse Simpson diversity index (Pearson = − 0.786, *p* = 0.036), Simpson diversity index (Pearson = − 0.967, *p* = 0.001) and Pielou evenness (Pearson = − 0.981, *p* = 0.001). For phylogenetic diversity, disease index significantly decreased with higher NTI (Pearson = − 0.866, *p* = 0.012) and MPD (Pearson = − 0.78, *p* = 0.039). However, there were no significant relationships between disease index and MNTD (Pearson = − 0.668, *p* = 0.101) or NRI (Pearson = − 0.536, *p* = 0.214). Network properties, including nodes, links, average K and modularity, showed no significant (*p* > 0.050) correlation with disease index (Additional file 1: Table S4). Pearson correlation analysis (Additional file 1: Table S4) also showed that Shannon diversity index (Pearson = − 0.834, *p* = 0.020), Simpson diversity index (Pearson = − 0.874, *p* = 0.010), Pielou evenness (Pearson = − 0.892, *p* = 0.007) and MPD (Pearson = − 0.874, *p* = 0.010) were significantly and negatively correlated to the disease infection rate.

## Discussion

The relationship between the bacterial community and crop morbidity is an important topic in microbial ecology and biocontrol of crop disease (Xiao et al. 2018). Previous studies in biological control have mainly focused on soil bacterial community and revealed that soil bacterial community played a critical role in crop disease incidence (Wang et al. 2017; Yang et al. 2017). However, very few investigations have been conducted to address the potential interactions between introduced BCAs, indigenous microbial communities in the phyllosphere and crop health. As many foliar bacterial pathogens colonize plant surfaces before infection, the microbiology of the phyllosphere has been applied to the promotion of plant growth and plant protection recently (Vorholt 2012). Tobacco wildfire disease caused by *Pseudomonas syringae* pv*. tabaci* is the main leaf bacterial disease on tobacco. In this study, we investigated the responses of phyllosphere microbiota and plant health to application of two BCAs. The results showed that application of BCAs, especially BCA_B, could decrease the plant disease infection rate and disease index significantly. Phyllosphere bacterial community, including bacterial composition, taxonomic and phylogenetic diversity, community structure, and microbial interactions, showed great changes by application of BCAs. Through further analyses, it was found that community diversity of the phyllosphere was negatively correlated to disease infection rate and the disease index.

Until now, BCAs have shown effectiveness and have been successfully employed in the pest and disease management programs (Chen et al. 2009). A number of studies have reported that BCAs have potential in biocontrol of plant pathogens and promoting plant growth (Ren et al. 2012; Han et al. 2016). Consistent results were obtained in the present study. The two BCAs used in our study had remarkable control effects against tobacco wildfire disease, but showed still some disparity of performance in antagonistic efficiency (Fig. 1), indicating that the same plant responds variously to different BCAs. Compared to inoculation into soil, spraying BCAs on plant leaves cannot change the soil physicochemical properties, thus the most likely mechanism in inhibiting pathogen by BCAs is changing microbial community in the phyllosphere. However, many studies demonstrated that phyllosphere microbiota in field conditions were not greatly affected by the introduced BCAs (Perazzolli et al. 2014; Wei et al. 2016), which were contrary to our results that application of BCAs changed phyllosphere microbial community significantly at both diversity and composition (Table 1, Fig. 3 and Additional file 1: Fig. S4). The differences might be attributed to the traits of BCAs and application methods. On the one hand, the BCAs used in our study were mixed cultures, which might cause greater disturbances on an indigenous community than inoculation of a single strain. The phenomenon was consistent with the findings that co-inoculation of biocontrol agents caused a more pronounced impact on the microbial community structure than single application (Grosch et al. 2012). Multiple populations of biocontrol bacteria might contribute additively to biocontrol in situ (Kim et al. 2011). On the other hand, the numbers of applied BCAs usually rapidly decline after introduction to the phyllosphere because of the harsh environment (Longa et al. 2008). Therefore, repeated application of BCAs on leaves might increase the chances to maintain and build up an active population (Wei et al. 2016).

According to the difference analyses, treatments with BCAs significantly affected the indigenous bacterial community on tobacco leaves. Although bacteria belonging to the *Proteobacteria* phylum predominated on both treated and untreated tobacco leaves, the genus composition of the phyllosphere in different treatments was apparently different with time. After repeated spray of BCAs (at Day 28), the genera of *Sphingomonas* and *Pantoea* were significantly higher in BCA treatments than control (Additional file 1: Fig. S4). These taxa are often considered as plant-beneficial microbes (Enya et al. 2007a). Members of the genus *Pantoea* are frequently isolated from a wide range of ecological niches and have various biological roles, as plant epi- or endophytes, biocontrol agents or plant-growth promoters (De Maayer et al. 2012). Some *Pantoea* species can produce *N*-acyl-homoserine lactone (AHL) and the plant-growth hormone indole-3-acetic acid (IAA) (Enya et al. 2007b), fix nitrogen from the atmosphere (Loiret et al. 2004) and establish quorum sensing systems on leaves, which makes them possible to suppress pathogens on leaves (Frances et al. 2006; Pusey et al. 2011). *Sphingomonas* spp. is a Gram-negative, rod-shaped aerobic bacterium that is a highly competitive plant leaf colonizer. In a series of experiments, researchers demonstrated that the leaf bacterium *Sphingomonas* spp. could protect plants against the leaf-pathogenic *Pseudomonas syringae* through substrate competition (Innerebner et al. 2011). Carbon partitioning plays an important role for *Sphingomonas* spp. to be effective antagonists in the phyllosphere (Delmotte et al. 2009). Also, *Sphingomonas* spp. can promote agriculturally important crops growth by production of plant growth-stimulating factors (Enya et al. 2007b).

Many studies have indicated that an increase of soil bacterial diversity and control of some bacterial abundances could be an effective approach in controlling plant disease incidence (van Elsas et al. 2012; Yang et al. 2017; Xiao et al. 2018). Here, our study showed the phyllosphere community diversity, both taxonomic and phylogenetic diversity, was associated with tobacco wildfire disease infection and disease index (Fig. 6 and Additional file 1: Table S4). Taxonomic diversity (Shannon index, Inverse Simpson index, Simpson index and Pielou evenness) and phylogenetic diversity (near taxon index and mean phylogenetic distance) were more diverse in fields with healthy plants than in fields with infection. This is in line with a recent report in soil that microbial community diversity was positively correlated with plant health and a more diverse microbial community was beneficial for tobacco wilt suppression (Yang et al. 2017). Studies on soil communities also suggested that elevated levels in diversity and species richness contribute to high functional redundancy within the microbiome and thus could make it possible to quickly recover during stress and confer protection against soil-borne disease (Zak et al. 2003; Mendes et al. 2011). This mechanism might also be applicable to the microbial community in the phyllosphere. In addition, the resources on plants’ surface are exceedingly poor for phyllosphere microorganisms. Under limited conditions, diverse communities compete for resources more intensely than simple ones (Mallon et al. 2015, 2016), which could prevent bacterial pathogen invasion and subsequent growth (Jousset et al. 2011; van Elsas et al. 2012). Therefore, a diverse microbial community has in theory a higher probability of containing antagonists to pathogens or higher antagonistic co-evolutionary potential (Kinkel et al. 2011).

Different species/populations in natural ecosystem interact with each other to group as antagonistic, competitive or mutualistic networks (Olesen et al. 2007). In the present study, we further explored the bacterial interactions in the phyllosphere during the plant growth period using the RMT, which is powerful for identifying molecule ecological networks in microbial communities and has previously been employed to analyze the co-occurrence/interaction among different microbial populations (Zhou et al. 2011; Deng et al. 2012). Even though the correlation between the network indexes and wildfire infection was not strong (Fig. 6 and Additional file 1: Table S4), our results showed that bacterial interactions in BCA treatments (especially for BCA_B) were more connected and complex than that in the control (Additional file 1: Table S3 and Fig. 5). The network structure of the control group became simpler with plant growth, whereas the network complexity increased by application of BCA_B with time (Fig. 5). Many studies have shown that a complex microbial network was often less prone to pathogens invasion than a simpler one (Yang et al. 2017; Xiao et al. 2018). More complicated networks could be able to cope with the diverse and complex environmental changes (Berry and Widder 2014; Tao et al. 2018). At the same time, high interactions within communities could increase competition by leading to generally more efficient consumption of resources, and hence decreased the colonization success of pathogens (van Elsas et al. 2012). Therefore, the increased microbial interactions in the phyllosphere might also establish a “spatial repellent barrier” to against invasive pathogens.

In summary, the present study showed the effects of two different BCAs on the phyllosphere microbial community and further revealed the potential relationships between phyllosphere bacterial community and plant health. We found that (i) BCAs used in our study had remarkable control effects against tobacco wildfire disease, but showed still some disparity of performance in antagonistic efficiency; (ii) phyllosphere microbial community, including community diversity, taxonomic composition and microbial interactions, changed significantly after application of BCAs; (iii) phyllosphere microbial diversity was negatively correlated to tobacco wildfire disease infection. According to the inferred molecular ecology networks, we found that a more complex network might be beneficial for decreasing the chances of bacterial wildfire outbreak, and the genera of *Pantoea* and *Sphingomonas* may play important roles in wildfire disease suppression.

## Additional file

**Additional file 1.** Additional figures and table.

## References

[CR1] Akhtar MS, Siddiqui ZA (2010). Role of plant growth promoting rhizobacteria in biocontrol of plant diseases and sustainable agriculture. Microbiol Monogr.

[CR2] Alamri SAM, Hashem M, Mostafa YS, Nafady NA, Abo-Elyousr KAM (2019). Biological control of root rot in lettuce caused by *Exserohilum rostratum* and *Fusarium oxysporum* via induction of the defense mechanism. Biol Control.

[CR3] Bastian M, Heymann S, Jacomy M (2009). Gephi: an open source software for exploring and manipulating networks. ICWSM.

[CR4] Berry D, Widder S (2014). Deciphering microbial interactions and detecting keystone species with co-occurrence networks. Front Microbiol.

[CR5] Caporaso JG, Lauber CL, Walters WA, Berg-Lyons D, Huntley J, Fierer N, Owens SM, Betley J, Fraser L, Bauer M (2012). Ultra-high-throughput microbial community analysis on the Illumina HiSeq and MiSeq platforms. ISME J.

[CR6] Cha JY, Han S, Hong HJ, Cho H, Kim D, Kwon Y, Kwon SK, Crüsemann M, Yong BL, Kim JF (2016). Microbial and biochemical basis of a *Fusarium* wilt-suppressive soil. ISME J.

[CR7] Chen XH, Koumoutsi A, Scholz R, Schneider K, Vater J, Sussmuth R, Piel J, Borriss R (2009). Genome analysis of *Bacillus amyloliquefaciens* FZB42 reveals its potential for biocontrol of plant pathogens. J Biotechnol.

[CR8] De Maayer P, Chan W-Y, Blom J, Venter SN, Duffy B, Smits THM, Coutinho TA (2012). The large universal *Pantoea* plasmid LPP-1 plays a major role in biological and ecological diversification. BMC Genomics.

[CR9] Delmotte N, Knief C, Chaffron S, Innerebner G, Roschitzki B, Schlapbach R, von Mering C, Vorholt JA (2009). Community proteogenomics reveals insights into the physiology of phyllosphere bacteria. P Natl Acad Sci.

[CR10] Deng Y, Jiang YH, Yang Y, He Z, Luo F, Zhou J (2012). Molecular ecological network analyses. BMC Bioinf.

[CR11] Deng Y, Zhang P, Qin Y, Tu Q, Yang Y, He Z, Schadt CW, Zhou J (2016). Network succession reveals the importance of competition in response to emulsified vegetable oil amendment for uranium bioremediation. Environ Microbiol.

[CR12] Dixon P (2003). VEGAN, a package of R functions for community ecology. J Veg Sci.

[CR13] Edgar RC (2013). UPARSE: highly accurate OTU sequences from microbial amplicon reads. Nat Methods.

[CR14] Enya J, Koitabashi M, Shinohara H, Yoshida S, Tsukiboshi T, Negishi H, Suyama K, Tsushima S (2007). Phylogenetic diversities of dominant culturable *Bacillus*, *Pseudomonas* and *Pantoea* species on tomato leaves and their possibility as biological control agents. J Phytopathol.

[CR15] Enya J, Shinohara H, Yoshida S, Negishi TTH, Suyama K, Tsushima S (2007). Culturable leaf-associated bacteria on tomato plants and their potential as biological control agents. Microb Ecol.

[CR16] Erwin DC, Ribeiro OK (1996). *Phytophthora* diseases worldwide.

[CR17] Frances J, Bonaterra A, Moreno MC, Cabrefiga J, Badosa E, Montesinos E (2006). Pathogen aggressiveness and postharvest biocontrol efficiency in *Pantoea agglomerans*. Postharvest Biol Tec.

[CR18] Grosch R, Dealtry S, Schreiter S, Berg G, Mendonca-Hagler L, Smalla K (2012). Biocontrol of *Rhizoctonia solani*: complex interaction of biocontrol strains, pathogen and indigenous microbial community in the rhizosphere of lettuce shown by molecular methods. Plant Soil.

[CR19] Han T, You C, Zhang L, Feng C, Zhang C, Wang J, Kong F (2016). Biocontrol potential of antagonist *Bacillus subtilis* Tpb55 against tobacco black shank. Biocontrol.

[CR20] Innerebner G, Knief C, Vorholt JA (2011). Protection of *Arabidopsis thaliana* against leaf-pathogenic *Pseudomonas syringae* by *Sphingomonas* strains in a controlled model system. Appl Environ Microb.

[CR21] Janvier C, Villeneuve F, Alabouvette C, Edel-Hermann V, Mateille T, Steinberg C (2007). Soil health through soil disease suppression: which strategy from descriptors to indicators?. Soil Biol Biochem.

[CR22] Jensen B, Knudsen IMB, Andersen B, Nielsen KF, Thrane U, Jensen DF, Larsen J (2013). Characterization of microbial communities and fungal metabolites on field grown strawberries from organic and conventional production. Int J Food Microbiol.

[CR23] Jones JB, Vallad GE, Iriarte FB, Obradović A, Wernsing MH, Jackson LE, Balogh B, Hong JC, Momol MT (2012). Considerations for using bacteriophages for plant disease control. Bacteriophage.

[CR24] Jousset A, Schulz W, Scheu S, Eisenhauer N (2011). Intraspecific genotypic richness and relatedness predict the invasibility of microbial communities. ISME J.

[CR25] Kim YC, Leveau JHJ, Gardener BBM, Pierson EA, Pierson LS, Ryu CM (2011). The multifactorial basis for plant health promotion by plant-associated bacteria. Appl Environ Microb.

[CR26] Kinkel LL, Bakker MG, Schlatter DC (2011). A coevolutionary framework for managing disease-suppressive soils. Annu Rev Phytopathol.

[CR27] Leff JW, Fierer N (2013). Bacterial communities associated with the surfaces of fresh fruits and vegetables. PLoS ONE.

[CR28] Loiret FG, Ortega E, Kleiner D, Ortega-Rodes P, Rodes R, Dong Z (2004). A putative new endophytic nitrogen-fixing bacterium *Pantoea* sp. from sugarcane. J Appl Microbiol.

[CR29] Longa CMO, Pertot I, Tosi S (2008). Ecophysiological requirements and survival of a *Trichoderma atroviride* isolate with biocontrol potential. J Basic Microb.

[CR30] Lozupone C, Lladser ME, Dan K, Stombaugh J, Knight R (2011). UniFrac: an effective distance metric for microbial community comparison. ISME J.

[CR31] Magoc T, Salzberg SL (2011). FLASH: fast length adjustment of short reads to improve genome assemblies. Bioinformatics.

[CR32] Mallon Alexander C, Van Elsas JD, Salles Falcão J (2015). Microbial invasions: the process, patterns, and mechanisms. Trends Microbiol.

[CR33] Mallon CA, Poly F, Roux XL, Marring I, Elsas JDV, Salles JF (2016). Resource pulses can alleviate the biodiversity-invasion relationship in soil microbial communities. Ecology.

[CR34] Mendes R, Kruijt M, de Bruijn I, Dekkers E, van der Voort M, Schneider JHM, Piceno YM, DeSantis TZ, Andersen GL, Bakker PAHM, Raaijmakers JM (2011). Deciphering the rhizosphere microbiome for disease-suppressive bacteria. Science.

[CR35] Meyer KM, Leveau JHJ (2012). Microbiology of the phyllosphere: a playground for testing ecological concepts. Oecologia.

[CR36] Mueller T, Ruppel S (2014). Progress in cultivation-independent phyllosphere microbiology. FEMS Microbiol Ecol.

[CR37] Olesen JM, Bascompte J, Dupont YL, Jordano P (2007). The modularity of pollination networks. P Natl Acad Sci.

[CR38] Perazzolli M, Antonielli L, Storari M, Puopolo G, Pancher M, Giovannini O, Pindo M, Pertot I (2014). Resilience of the natural phyllosphere microbiota of the grapevine to chemical and biological pesticides. Appl Environ Microb.

[CR39] Pusey PL, Stockwell VO, Reardon CL, Smits THM, Duffy B (2011). Antibiosis activity of *Pantoea agglomerans* biocontrol strain E325 against *Erwinia amylovora* on apple flower stigmas. Phytopathology.

[CR40] Reed SC, Townsend AR, Cleveland CC, Nemergut DR (2010). Microbial community shifts influence patterns in tropical forest nitrogen fixation. Oecologia.

[CR41] Ren XL, Zhang N, Cao MH, Wu K, Shen QR, Huang QW (2012). Biological control of tobacco black shank and colonization of tobacco roots by a *Paenibacillus polymyxa* strain C5. Biol Fert Soils.

[CR42] Rouse DI, Nordheim EV, Hirano SS, Upper CD (1985). A model relating the probability of foliar disease incidence to the population frequencies of bacterial plant pathogens. Phytopathology.

[CR43] Sang MK, Kim KD (2012). Plant growth-promoting rhizobacteria suppressive to Phytophthora blight affect microbial activities and communities in the rhizosphere of pepper (*Capsicum annuum* L.) in the field. Appl Soil Ecol.

[CR44] Santhanam R, Van Thi L, Weinhold A, Goldberg J, Oh Y, Baldwin IT (2015). Native root-associated bacteria rescue a plant from a sudden-wilt disease that emerged during continuous cropping. P Natl Acad Sci.

[CR45] Sharma PS, D’Souza F, Kutner W (2012). Molecular imprinting for selective chemical sensing of hazardous compounds and drugs of abuse. Trac Trend Anal Chem.

[CR46] Sylla J, Alsanius BW, Krger E, Reineke A, Bischoff-Schaefer M, Wohanka W (2013). Introduction of *Aureobasidium pullulans* to the phyllosphere of organically grown strawberries with focus on its establishment and interactions with the resident microbiome. Agronomy.

[CR47] Sylla J, Alsanius BW, Krueger E, Reineke A, Strohmeier S, Wohanka W (2013). Leaf microbiota of strawberries as affected by biological control agents. Phytopathology.

[CR48] Tao J, Liu X, Liang Y, Niu J, Xiao Y, Gu Y, Ma L, Meng D, Zhang Y, Huang W (2016). Maize growth responses to soil microbes and soil properties after fertilization with different green manures. Appl Microbiol Biothnol.

[CR49] Tao J, Meng D, Qin C, Liu X, Liang Y, Xiao Y, Liu Z, Gu Y, Li J, Yin H (2018). Integrated network analysis reveals the importance of microbial interactions for maize growth. Appl Microbiol Biothnol.

[CR50] Trabelsi D, Mhamdi R (2013). Microbial inoculants and their impact on soil microbial communities: a review. Biomed Res Int.

[CR51] van Elsas JD, Chiurazzi M, Mallon CA, Elhottova D, Kristufek V, Salles JF (2012). Microbial diversity determines the invasion of soil by a bacterial pathogen. P Natl Acad Sci.

[CR52] Venkategowda R, Muthappa SK, Yasuhiro I, Amita K, Makarla U, Mysore KS (2013). Drought stress acclimation imparts tolerance to *Sclerotinia sclerotiorum* and *Pseudomonas syringae* in *Nicotiana benthamiana*. Int J Mol Sci.

[CR53] Vorholt JA (2012). Microbial life in the phyllosphere. Nat Rev Microbiol.

[CR54] Wang Q, Garrity GM, Tiedje JM, Cole JR (2007). Naïve bayesian classifier for rapid assignment of rRNA sequences into the new bacterial taxonomy. Appl Environ Microb.

[CR55] Wang R, Zhang H, Sun L, Qi G, Chen S, Zhao X (2017). Microbial community composition is related to soil biological and chemical properties and bacterial wilt outbreak. Sci Rep.

[CR56] Webb CO, Ackerly DD, McPeek MA, Donoghue MJ (2002). Phylogenies and community ecology. Annu Rev Ecol Syst.

[CR57] Wei F, Hu X, Xu X (2016). Dispersal of *Bacillus subtilis* and its effect on strawberry phyllosphere microbiota under open field and protection conditions. Sci Rep.

[CR58] Williams TR, Moyne A-L, Harris LJ, Marco ML (2013). Season, irrigation, leaf age, and *Escherichia coli* inoculation influence the bacterial diversity in the lettuce phyllosphere. PLoS ONE.

[CR59] Xiao Y, Liu X, Meng D, Tao J, Gu Y, Yin H, Li J (2018). The role of soil bacterial community during winter fallow period in the incidence of tobacco bacterial wilt disease. Appl Microbiol Biot.

[CR60] Yang H, Li J, Xiao Y, Gu Y, Liu H, Liang Y, Liu X, Hu J, Meng D, Yin H (2017). An integrated insight into the relationship between soil microbial community and tobacco bacterial wilt disease. Front Microbiol.

[CR61] You C, Zhang C, Feng C, Wang J, Kong F (2015). *Myroides odoratimimus*, a biocontrol agent from the rhizosphere of tobacco with potential to control *Alternaria alternata*. Biocontrol.

[CR62] Zak DR, Holmes WE, White DC, Peacock AD, Tilman D (2003). Plant diversity, soil microbial communities, and ecosystem function: are there any links?. Ecology.

[CR63] Zhang B, Bai Z, Hoefel D, Tang L, Yang Z, Zhuang G, Yang J, Zhang H (2008). Assessing the impact of the biological control agent *Bacillus thuringiensis* on the indigenous microbial community within the pepper plant phyllosphere. FEMS Microbiol Lett.

[CR64] Zhou J, Deng Y, Luo F, He Z, Tu Q, Zhi X (2010). Functional molecular ecological networks. mBio.

[CR65] Zhou J, Deng Y, Luo F, He Z, Yang Y (2011). Phylogenetic molecular ecological network of soil microbial communities in response to elevated CO_2_. mBio.

