# Supplementary material for: Responses of phyllosphere microbiota and plant health to application of two different biocontrol agents
Source: AMB Express. 2019 Mar 28;9:42. doi: 10.1186/s13568-019-0765-x (PMC6439047; doi:10.1186/s13568-019-0765-x)
Supplement: Supplementary file 1 — Additional file 1. Additional figures and table. [file 13568_2019_765_MOESM1_ESM.docx]

*Additional file*

**Responses of phyllosphere microbiota and plant health to application of two different biocontrol agents**

Chong Qin^1,2^, Jiemeng Tao^1,2^, Tianbo Liu^3^, Yongjun Liu^4^, Nengwen Xiao^5^, Tianming Li^1,2^, Yabing Gu^1,2^, Huaqun Yin^1,2^ and Delong Meng^1,2^^*^

^*^ Corresponding author: Delong Meng

^1^ School of Minerals Processing and Bioengineering, Central South University, Changsha, China.

^2^ Key Laboratory of Biometallurgy, Ministry of Education, Central South University, Changsha, China.

^3^ Tobacco Research Institute of Hunan Province, Changsha, China

^4^ College of Agronomy, Hunan Agricultural University, Changsha, China

^5^ State Key Laboratory of Environmental Criteria and Risk Assessment, Chinese Research Academy of Environmental Sciences, Beijing China

^*^Correspondence:

Delong Meng: [Delong.meng@gmail.com](mailto:Delong.meng@gmail.com)

Tel: +86(731)88830546

Fax: +86(731)88830546

# Additional Figures


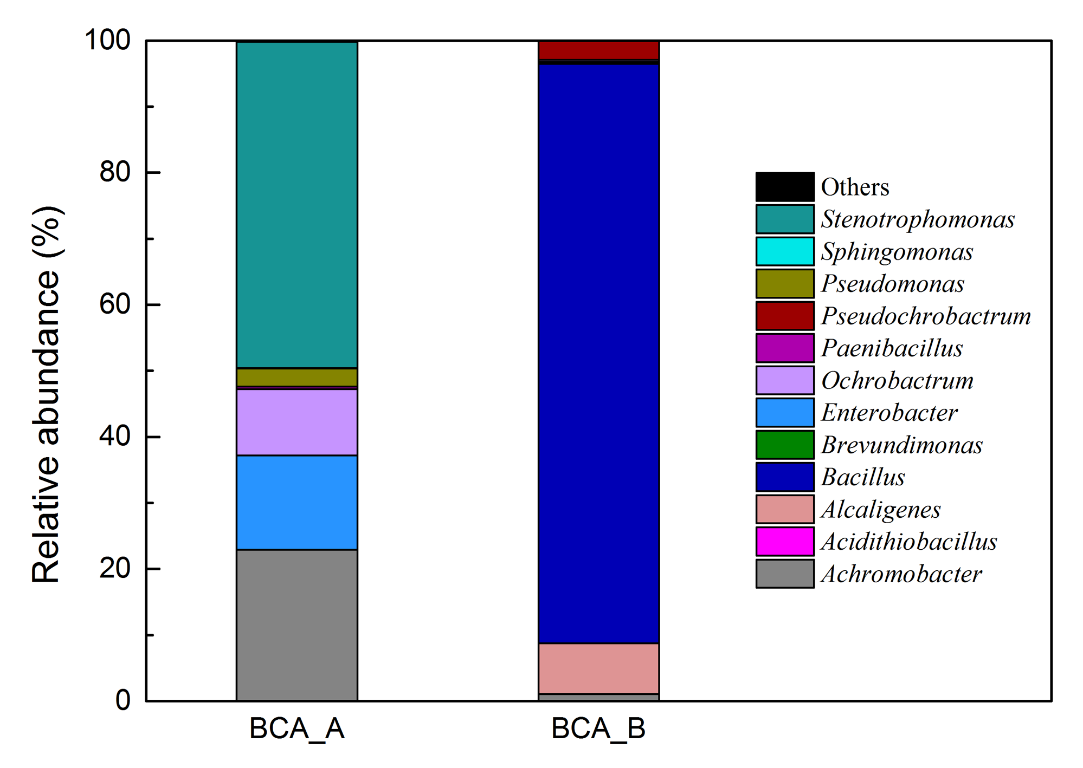


**Figure S1.** Microbial composition of biocontrol agents (BCAs) at genus level. The genera whose relative abundance is less than 0.1% were defined as “Others”. The sequencing data of the agents have been made publicly available in the Sequence Read Archive (SRA) database of NCBI following the accession number of PRJNA515831.


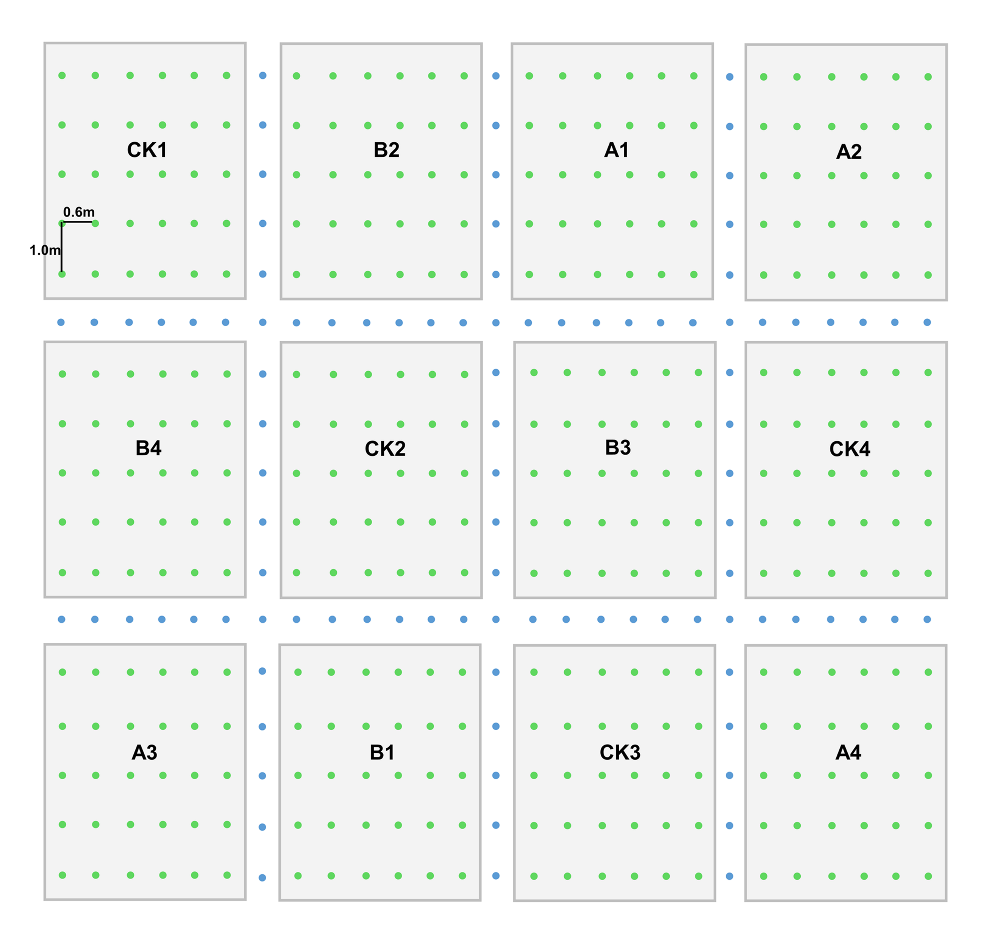


**Figure S2.** Experiment design and overall arrangement of treatments in this study. Tobacco plants were transplanted to a field with a strain spacing of 0.6 m and a row spacing of 1.0 m on May 10^th^ 2017. The green dots represent the tested plants and the blue dots represent plants in guard rows. The experiment included three treatments (control group, application of biocontrol agent A and application of biocontrol agent B), which were arranged in a randomized complete block design with 12 plots. The agents were sprayed on tobacco leaves on June 28^th^, June 5^th^, July 12^th^ and July 19^th^, respectively. During the experiment, disease infection rate and disease index of wildfire disease were recorded, and tobacco leaves were collected.


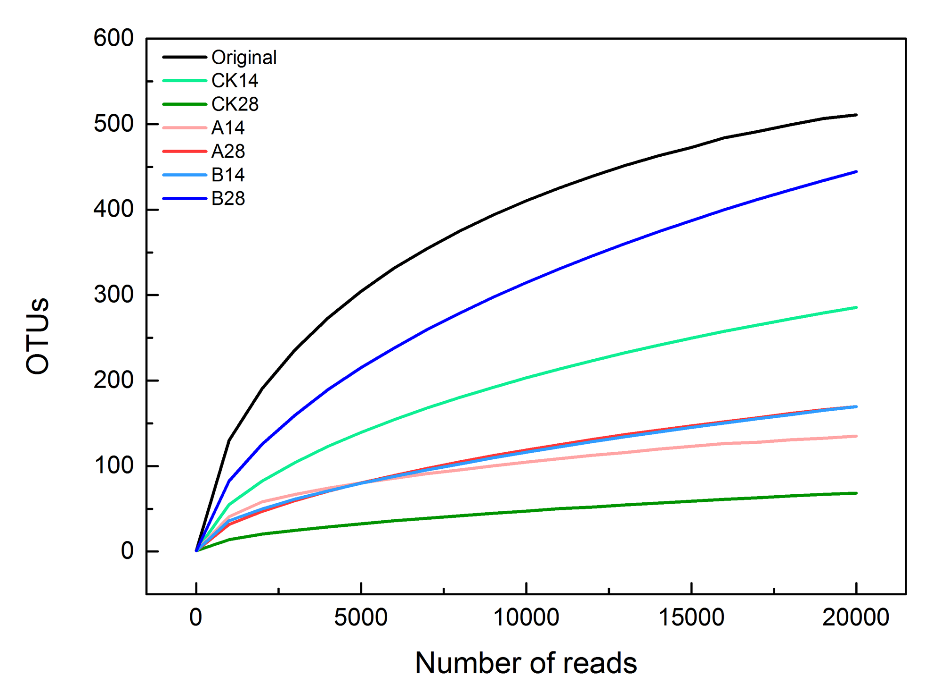


**Figure S3.** Rarefaction curve of 16S rRNA gene sequencing in each treatment. ‘Original’ represents samples collected before application of biocontrol agents. Abbreviations of CK, A and B represent the control group and tobacco leaves sprayed with biocontrol agent (BCA) A and biocontrol agent B, respectively. The number 14 and 28 represent the time (days) after BCAs application.


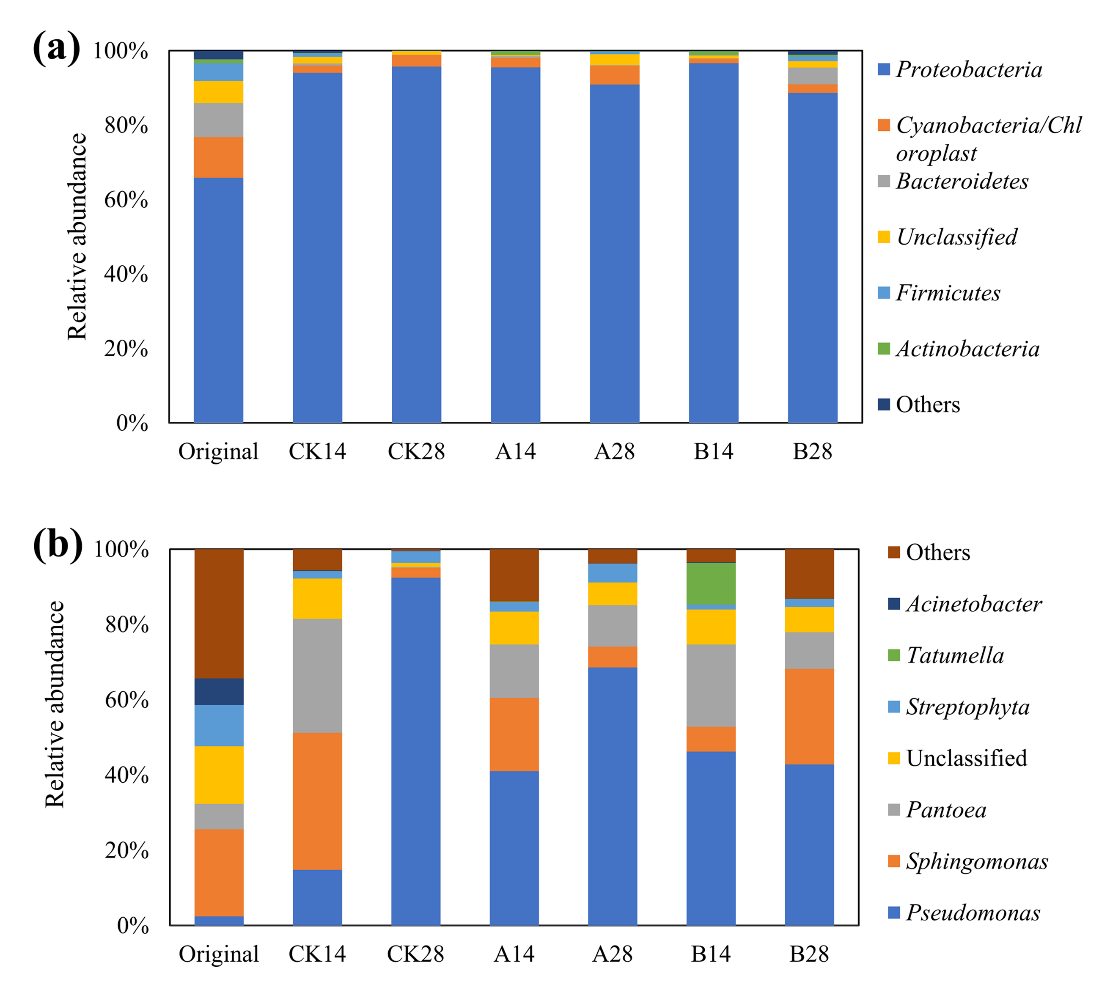


**Figure S4.** Phylogenetic tree of major OTUs and microbial composition at phylum level (a) and genus level (b) on tobacco leaves by application of biocontrol agents. ‘Original’ represents samples collected before application of biocontrol agents. Abbreviations of CK, A and B represent the control group and tobacco leaves sprayed with biocontrol agent (BCA) A and biocontrol agent B, respectively. The number 14 and 28 represent the time (days) after BCAs application.

# Additional Tables

**Table S1.** Significance tests of the effects of biocontrol agents (BCAs) on the taxonomic characteristics of phyllosphere microbial community structure.

|  |  | MRPP | | ANOSIM | | ADONIS | |
| --- | --- | --- | --- | --- | --- | --- | --- |
| Group | Treatment | δ | *P* | R | *P* | F | *P* |
| Original vs Day 14 |  | 0.586 | 0.001 | 0.543 | 0.001 | 6.674 | 0.001 |
| Original vs Day 28 |  | 0.62 | 0.001 | 0.238 | 0.013 | 4.803 | 0.002 |
| Day 14 vs Day 28 |  | 0.638 | 0.002 | 0.141 | 0.003 | 2.771 | 0.002 |
| Control vs BCA_A |  | 0.604 | 0.001 | 0.324 | 0.001 | 3.768 | 0.001 |
|  | CK14 vs A14 | 0.499 | 0.002 | 0.967 | 0.002 | 8.846 | 0.001 |
|  | CK28 vs A28 | 0.524 | 0.002 | 0.958 | 0.001 | 5.158 | 0.001 |
| Control vs BCA_ B |  | 0.637 | 0.002 | 0.211 | 0.008 | 3.136 | 0.001 |
|  | CK14 vs B14 | 0.556 | 0.001 | 0.636 | 0.001 | 3.309 | 0.001 |
|  | CK28 vs B28 | 0.553 | 0.001 | 1 | 0.001 | 9.298 | 0.001 |
| BCA_ A vs BCA_ B |  | 0.602 | 0.001 | 0.261 | 0.001 | 3.955 | 0.001 |
|  | A14 vs B14 | 0.488 | 0.001 | 0.867 | 0.001 | 5.518 | 0.001 |
|  | A28 vs B28 | 0.546 | 0.001 | 0.713 | 0.001 | 5.036 | 0.001 |

Three different permutation tests were performed, including the multiple response permutation procedure (MRPP), analysis of similarity (ANOSIM) and permutational multivariate analysis of variance (Adonis), calculated with Bray-curtis distance. *p* value represents corresponding significance test. ‘Original’ represents samples collected before application of biocontrol agents. Abbreviations of CK, A and B represent the control group and tobacco leaves sprayed with biocontrol agent (BCA) A and biocontrol agent B, respectively. The number 14 and 28 represent the time (days) after BCAs application.

**Table S2** Significance tests of the effects of biocontrol agents on the phylogenetic characteristics of phyllosphere microbial community structure.

|  |  | MRPP | | ANOSIM | | ADONIS | |
| --- | --- | --- | --- | --- | --- | --- | --- |
| Group | Treatment | δ | *p* | R | *p* | F | *p* |
| Original vs Day 14 |  | 0.335 | 0.001 | 0.718 | 0.001 | 13.682 | 0.001 |
| Original vs Day 28 |  | 0.311 | 0.001 | 0.819 | 0.001 | 19.188 | 0.001 |
| Day 14 vs Day 28 |  | 0.299 | 0.001 | 0.221 | 0.001 | 6.427 | 0.005 |
| Control vs BCA_ A |  | 0.329 | 0.109 | 0.059 | 0.107 | 0.643 | 0.564 |
|  | CK14 vs A14 | 0.29 | 0.006 | 0.209 | 0.03 | 3.046 | 0.08 |
|  | CK28 vs A28 | 0.128 | 0.002 | 0.399 | 0.002 | 4.963 | 0.001 |
| Control vs BCA_ B |  | 0.335 | 0.068 | 0.041 | 0.138 | 1.216 | 0.307 |
|  | CK14 vs B14 | 0.23 | 0.001 | 0.338 | 0.003 | 8.079 | 0.003 |
|  | CK28 vs B28 | 0.183 | 0.001 | 0.748 | 0.001 | 18.493 | 0.001 |
| BCA_ A vs BCA_ B |  | 0.291 | 0.034 | 0.027 | 0.207 | 1.832 | 0.157 |
|  | A14 vs B14 | 0.185 | 0.006 | 0.482 | 0.008 | 5.091 | 0.026 |
|  | A28 vs B28 | 0.246 | 0.006 | 0.494 | 0.005 | 7.489 | 0.011 |

Three different permutation tests were performed, including the multiple response permutation procedure (MRPP), analysis of similarity (ANOSIM) and permutational multivariate analysis of variance (Adonis), calculated with Bray-curtis distance. *p* value represents corresponding significance test. ‘Original’ represents samples collected before application of biocontrol agents. Abbreviations of CK, A and B represent the control group and tobacco leaves sprayed with biocontrol agent (BCA) A and biocontrol agent B, respectively. The number 14 and 28 represent the time (days) after BCAs application.

**Table S3** The basic topological properties of the networks deriving from phyllosphere microbial communities in different treatments.

| Network | Similarity threshold (*St*) | Total nodes | Total links | Average degree (avgK) | Modularity |
| --- | --- | --- | --- | --- | --- |
| Original | 0.950 | 254 | 1746 | 13.748 | 0.551 |
| CK_14 | 0.860 | 145 | 533 | 7.352 | 0.577 |
| A_14 | 0.770 | 113 | 1707 | 30.212 | 0.091 |
| B_14 | 0.770 | 96 | 482 | 10.042 | 0.349 |
| CK_28 | 0.790 | 41 | 55 | 2.683 | 0.571 |
| A_28 | 0.770 | 119 | 602 | 10.118 | 0.430 |
| B_28 | 0.930 | 236 | 1414 | 11.983 | 0.628 |

‘Original’ represents samples collected before application of biocontrol agents. Abbreviations of CK, A and B represent the control group and tobacco leaves sprayed with biocontrol agent (BCA) A and biocontrol agent B, respectively. The number 14 and 28 represent the time (days) after BCAs application.

**Table S4** Pearson correlation matrix of sizes, which are related to plant health (wildfire disease) and phyllosphere microbial communities (taxonomic diversity, phylogenetic diversity and network properties). IR: infection rate; DI: disease index; Sob: observed OTU number; Chao1: Chao1 richness Index; H: Shannon diversity index; InvD: Inverse Simpson diversity index; D: Simpson diversity index; E: Pielou evenness; MNTD: mean-nearest-taxon-distance; NTI: near-taxon-index; MPD: mean phylogenetic distance; NRI: net relatedness index; agv K: average degree. *p*-values highlighted in bold show significance (*p* < 0.05).

|  | IR | DI | Sob | Chao1 | H | InvD | D | E | MNTD | NTI | MPD | NRI | Nodes | Links | avgK |
| --- | --- | --- | --- | --- | --- | --- | --- | --- | --- | --- | --- | --- | --- | --- | --- |
| DI | 0.921 |  |  |  |  |  |  |  |  |  |  |  |  |  |  |
|  | **0.003** |  |  |  |  |  |  |  |  |  |  |  |  |  |  |
| Sob | -0.333 | -0.494 |  |  |  |  |  |  |  |  |  |  |  |  |  |
|  | 0.465 | 0.26 |  |  |  |  |  |  |  |  |  |  |  |  |  |
| Chao1 | -0.314 | -0.493 | 0.963 |  |  |  |  |  |  |  |  |  |  |  |  |
|  | 0.493 | 0.261 | **0.001** |  |  |  |  |  |  |  |  |  |  |  |  |
| H | -0.834 | -0.947 | 0.717 | 0.694 |  |  |  |  |  |  |  |  |  |  |  |
|  | **0.02** | **0.001** | **0.07** | 0.084 |  |  |  |  |  |  |  |  |  |  |  |
| InvD | -0.738 | -0.786 | 0.641 | 0.504 | 0.858 |  |  |  |  |  |  |  |  |  |  |
|  | 0.058 | **0.036** | 0.121 | 0.249 | **0.014** |  |  |  |  |  |  |  |  |  |  |
| D | -0.874 | -0.967 | 0.479 | 0.541 | 0.918 | 0.643 |  |  |  |  |  |  |  |  |  |
|  | **0.01** | **0.001** | 0.277 | 0.21 | **0.004** | 0.119 |  |  |  |  |  |  |  |  |  |
| E | -0.892 | -0.981 | 0.513 | 0.514 | 0.964 | 0.782 | 0.968 |  |  |  |  |  |  |  |  |
|  | **0.007** | **0.001** | 0.239 | 0.238 | **0.001** | **0.038** | **0.001** |  |  |  |  |  |  |  |  |
| MNTD | -0.545 | -0.668 | 0.937 | 0.861 | 0.854 | 0.861 | 0.605 | 0.689 |  |  |  |  |  |  |  |
|  | 0.206 | 0.101 | **0.002** | **0.013** | **0.014** | **0.013** | 0.15 | 0.087 |  |  |  |  |  |  |  |
| NTI | -0.874 | -0.866 | 0.099 | 0.073 | 0.748 | 0.713 | 0.807 | 0.871 | 0.394 |  |  |  |  |  |  |
|  | **0.01** | **0.012** | 0.832 | 0.876 | 0.053 | 0.072 | **0.028** | **0.011** | 0.382 |  |  |  |  |  |  |
| MPD | -0.625 | -0.78 | 0.871 | 0.816 | 0.936 | 0.825 | 0.75 | 0.829 | 0.94 | 0.517 |  |  |  |  |  |
|  | 0.133 | **0.039** | **0.011** | **0.025** | **0.002** | **0.022** | 0.052 | **0.021** | **0.002** | 0.235 |  |  |  |  |  |
| NRI | -0.575 | -0.536 | -0.343 | -0.198 | 0.266 | 0.013 | 0.604 | 0.474 | -0.215 | 0.63 | -0.071 |  |  |  |  |
|  | 0.177 | 0.214 | 0.451 | 0.67 | 0.564 | 0.978 | 0.151 | 0.282 | 0.643 | 0.129 | 0.88 |  |  |  |  |
| Nodes | -0.337 | -0.546 | 0.981 | 0.95 | 0.765 | 0.645 | 0.542 | 0.584 | 0.928 | 0.166 | 0.918 | -0.299 |  |  |  |
|  | 0.46 | 0.204 | **0.001** | **0.001** | **0.045** | 0.118 | 0.209 | 0.169 | **0.003** | 0.722 | **0.004** | 0.515 |  |  |  |
| Links | -0.501 | -0.678 | 0.63 | 0.586 | 0.826 | 0.7 | 0.678 | 0.791 | 0.745 | 0.581 | 0.894 | -0.044 | 0.742 |  |  |
|  | 0.252 | 0.094 | 0.129 | 0.167 | **0.022** | **0.08** | 0.094 | **0.034** | 0.055 | 0.172 | **0.007** | 0.925 | 0.056 |  |  |
| avgK | -0.496 | -0.588 | 0.023 | 0.026 | 0.562 | 0.375 | 0.622 | 0.702 | 0.205 | 0.727 | 0.482 | 0.375 | 0.182 | 0.771 |  |
|  | 0.258 | 0.165 | 0.962 | 0.956 | 0.189 | 0.408 | 0.136 | 0.079 | 0.659 | 0.064 | 0.273 | 0.407 | 0.696 | **0.042** |  |
| Modularity | 0.391 | 0.392 | 0.503 | 0.48 | -0.195 | -0.097 | -0.399 | -0.434 | 0.273 | -0.71 | 0.027 | -0.67 | 0.367 | -0.282 | -0.805 |
|  | 0.386 | 0.384 | 0.25 | 0.276 | 0.675 | 0.836 | 0.376 | 0.33 | 0.554 | **0.074** | 0.954 | 0.1 | 0.418 | 0.539 | **0.029** |
